# Neuroprotective Effects of CGP3466B on Apoptosis Are Modulated by Protein-L-isoaspartate (D-aspartate) O-methyltransferase/Mst1 Pathways after Traumatic Brain Injury in Rats

**DOI:** 10.1038/s41598-017-08196-3

**Published:** 2017-08-23

**Authors:** Feng Liang, Ligen Shi, Jingwei Zheng, Sheng Chen, Yangxin Wang, Jianmin Zhang

**Affiliations:** 1 Department of Neurosurgery, Second Affiliated Hospital, School of Medicine, Zhejiang University, Hangzhou, 310009 China; 2 Department of Orthopaedics, Second Affiliated Hospital, School of Medicine, Zhejiang University, Hangzhou, 310009 China

## Abstract

Neuronal apoptosis chiefly contributes to the cell loss following traumatic brain injury (TBI). CGP3466B is a compound related to the anti-Parkinsonism drug R-(−)-deprenyl. Previous studies have illuminated anti-apoptosis effects of CGP3466B in different cell lines, but the underlying mechanisms have not been fully elucidated. Mammalian sterile 20 (STE20)-like kinase1 (Mst1) is a core component of the Hippo signaling pathway. Protein-L-isoaspartate (D-aspartate) O-methyltransferase (PCMT1) is an enzyme that repairs damaged L-isoaspartyl residues in proteins. The present study was performed to investigate the neuroprotective effects of CGP3466B and to determine a potential PCMT1/Mst1 neuronal anti-apoptotic pathway after TBI. Double immunofluorescence staining demonstrated that PCMT1 and Mst1 are co-located in neurons. Administration of CGP3466B improved neurological function, downregulated the ROS level and alleviated brain edema at 24 h after TBI. CGP3466B alleviates neuronal apoptosis by increasing PCMT1 expression and subsequently inhibiting MST1 activation, resulting in changing the expression levels of Bax, Bcl-2 and active-caspase3. The TUNEL staining results also support the anti-apoptosis effects of CGP3466B. The anti-apoptotic effects of CGP3466B were abolished by chelerythrine, an Mst1 activator, without changing PCMT1 levels. In conclusion, our findings suggest CGP3466B may have a promising therapeutic potential by modulating PCMT1/Mst1 signaling pathway after TBI injury.

## Introduction

Traumatic brain injury (TBI) has been a leading cause of death and disability among children and young adults worldwide^1^. There is no effective clinical treatment in part because of its complicated mechanisms. TBI-induced neural cell loss has been divided into primary and secondary events in previous studies^2, 3^. Primary impairment refers to the physical damage occurring at the time of trauma, whereas secondary impairment results from the metabolic and cellular changes induced by the insult^4^. The mechanisms of these secondary changes are rather complicated. Among them, neuronal apoptosis is very important to cell loss. However, the precise signaling pathway underlying the apoptosis process is still unclear.

Protein-L-isoaspartate (D-aspartate) O-methyltransferase (PCMT1) is an enzyme that repairs damaged L-isoaspartyl residues in proteins^5^. It is highly expressed in the brain, especially the neuron^6^. Recently, the anti-apoptotic properties of PCMT1have been explored in brain cells^7–9^, endothelial cells^10^ and cardiomyocytes *in vitro* ^11^. In addition, PCMT1 expression could have antioxidant properties against the reactive oxygen species (ROS) generated by cytosolic dopamine^8^.

Mammalian sterile 20 (STE20)-like kinase 1 (Mst1) is a multifunctional serine-threonine kinase, which is composed of an N-terminal catalytic domain and a non-catalytic C-terminal region^12^. As a core component of the Hippo signaling pathway, Mst1 is involved in a variety of regulatory mechanisms such as apoptosis, cell growth and stress response^13, 14^. In previous studies, Mst1 has been suggested to mediate neuronal cell apoptosis in Amyotrophic Lateral Sclerosis (ALS) and in cardiomyocytes apoptosis induced by hypoxia^11, 15^. During apoptosis, the activation of Mst1 occurs mainly through the release of C-terminal regulatory domain of Mst1, producing 34–36kDa N-terminal fragments by caspase-dependent cleavage^12^. This cleavage markedly increases Mst1 kinase activity translocating the cleaved-Mst1 to the nucleus to phosphorylate many substrates^16^. Several downstream pro-apoptotic targets such as histone H2B and FOXO have been recently identified^14, 16–18^. In addition, protein-protein interaction between Mst1 and PCMT1 has been identified in HEK293 cells^11^.

CGP3466B is a compound related to the anti-Parkinsonism drug R-(−)-deprenyl. Recent studies have illuminated anti-apoptosis effects of CGP3466B in neurons and cardiomyocytes cell lines^7, 11^. However, the underlying mechanisms still remained unclear. In the present study, we demonstrated the neuroprotective effects of CGP3466B and explored a possible PCMT1/Mst1 signaling pathway following TBI.

## Results

### Neurological Scores

**Figure 1:**
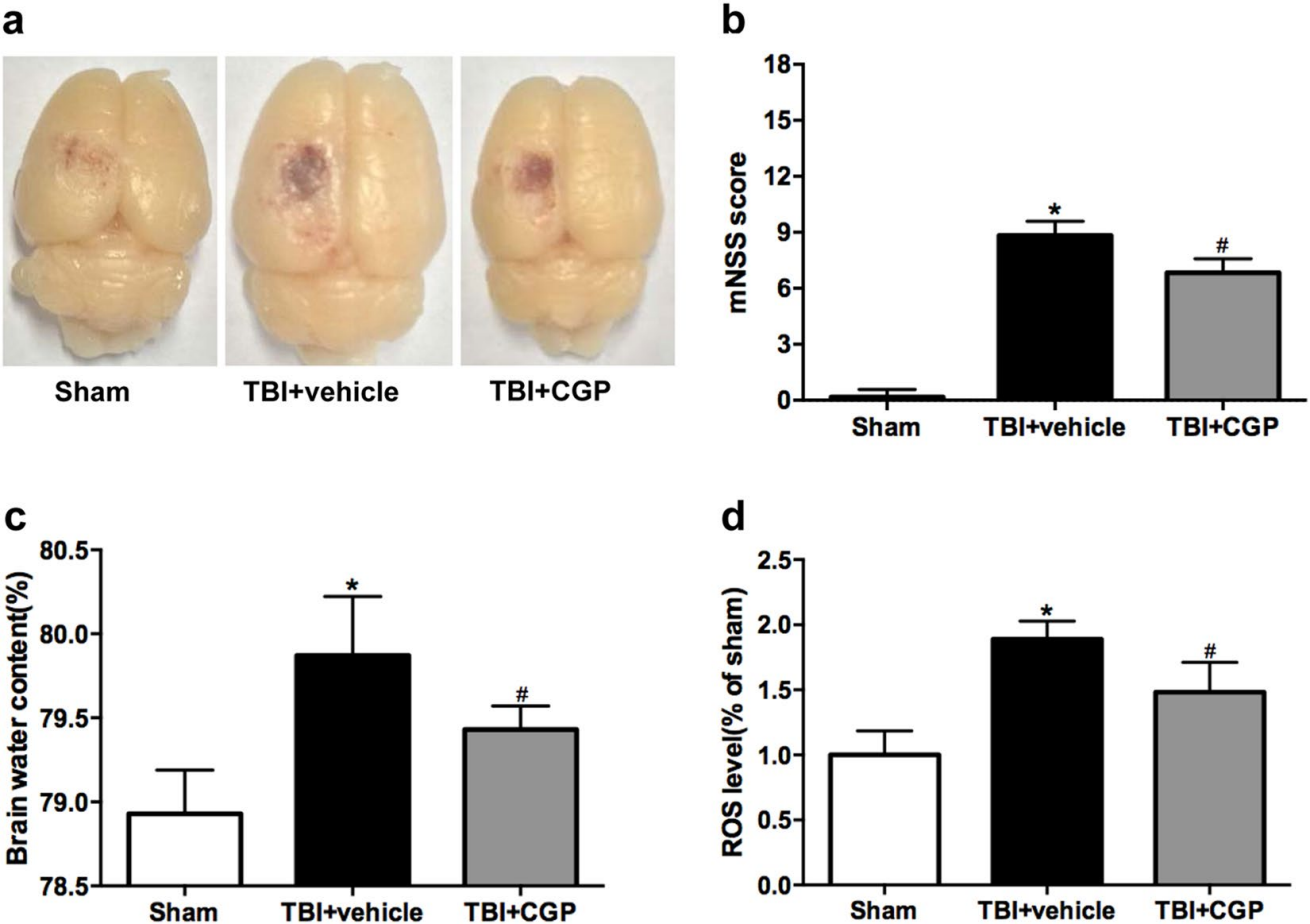
The representative pictures of the TBI model in different groups, the modiefied neurological severity scores (mNSS), the brain water content and ROS levels at 24 hours after TBI. (**a**) Typical brains from Sham, TBI+ vehicle, and TBI+ CGP group. (**b**) Quantification of the mNSS. (**c)** Quantification of the brain water content. (**d**) Quantification of ROS levels. Data are represented as the mean ± SD, n = 6 rats per group. * *P* < 0.05 vs sham, ^#^ *P* < 0.05 vs TBI+ vehicle.

mNss was measured at 24 h after surgery to confirm the effects of CGP3466B. As shown in Fig. 1b, the mNSS of TBI+ vehicle group was significantly higher than the sham group (*P* < 0.05). TBI animals that received CGP3466B showed improved neurological function (*P* < 0.05).

### CGP3466B Alleviated the Cerebral Edema

The brain water content of the injured hemisphere was significantly increased in the TBI+ vehicle group at 24 h after surgery. However, treatment with CGP3466B significantly alleviated the cerebral edema relative to the TBI+ vehicle group (*P* < 0.05, Fig. 1c).

### CGP3466B Downregulated the ROS Level

The ROS assay revealed that the cortical levels of ROS were significantly increased at 24 h after TBI, relative to the sham group (*P* < 0.05, Fig. 1d). Treatment with CGP3466B markedly reduced the ROS levels when compared with the TBI+ vehicle group (*P* < 0.05, Fig. 1d).

### PCMT1 and Mst1 Were Co-located in Neurons

**Figure 2:**
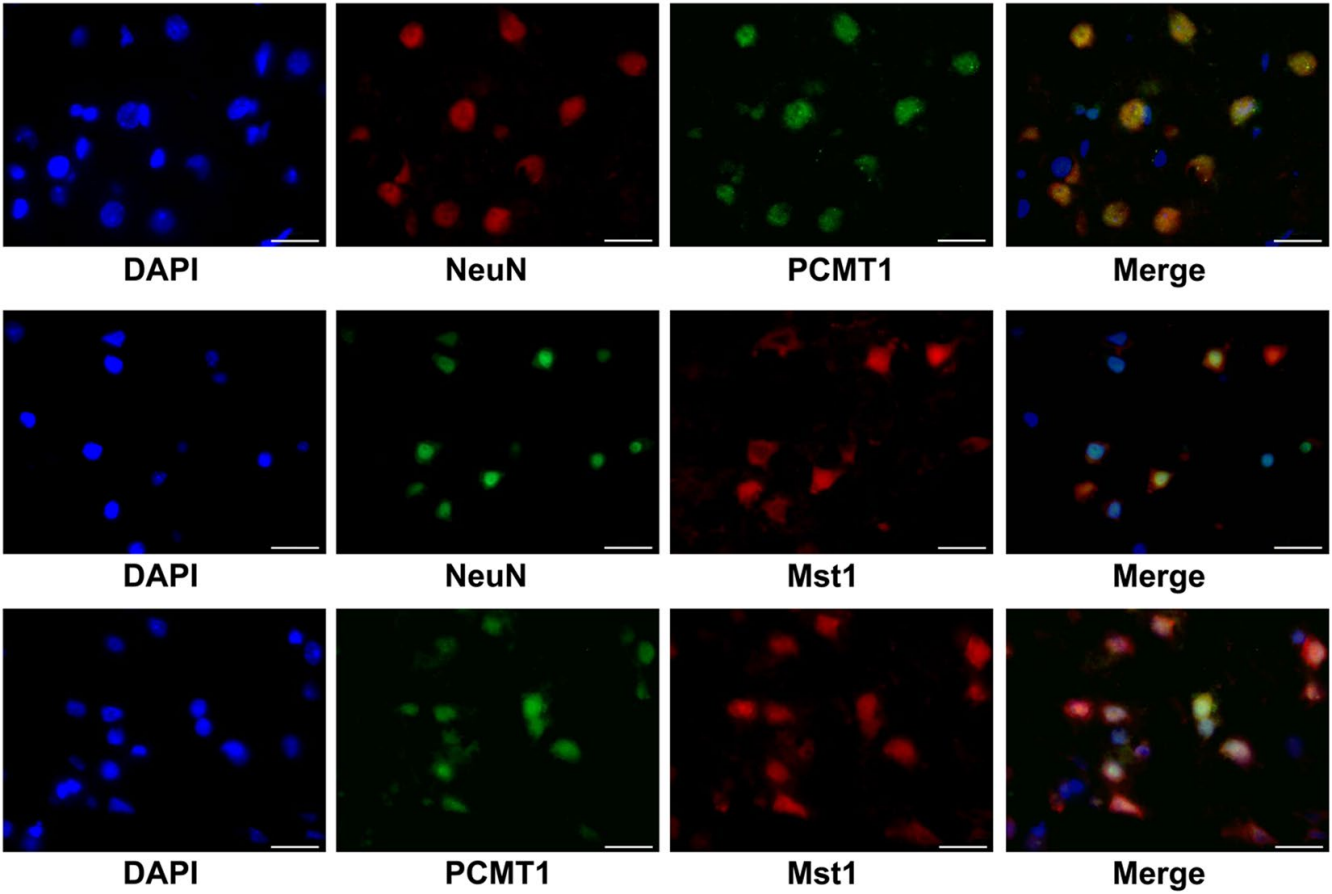
Double fluorescence labeling of PCMT1/Mst1/Neuron in peri-injured cortex at 24 h after TBI. PCMT1 and Mst1 were both co-located and highly expressed in the neurons. Scale bar = 20 μm.

We used double fluorescence labeling to identify the location of PCMT1 and Mst1 in TBI. Our results demonstrated that PCMT1 and Mst1 were mainly co-located in the neurons (Fig. 2).

### CGP3466B Upregulated PCMT1 Expression and Inhibited cleaved-Mst1 Activation After TBI

Western blot analysis was used to quantify the expression of PCMT1, Mst1, cleaved-Mst1, Bcl-2, Bax and active-caspase3 in the ipsilateral cerebral cortex 24 hours after injury.

**Figure 3:**
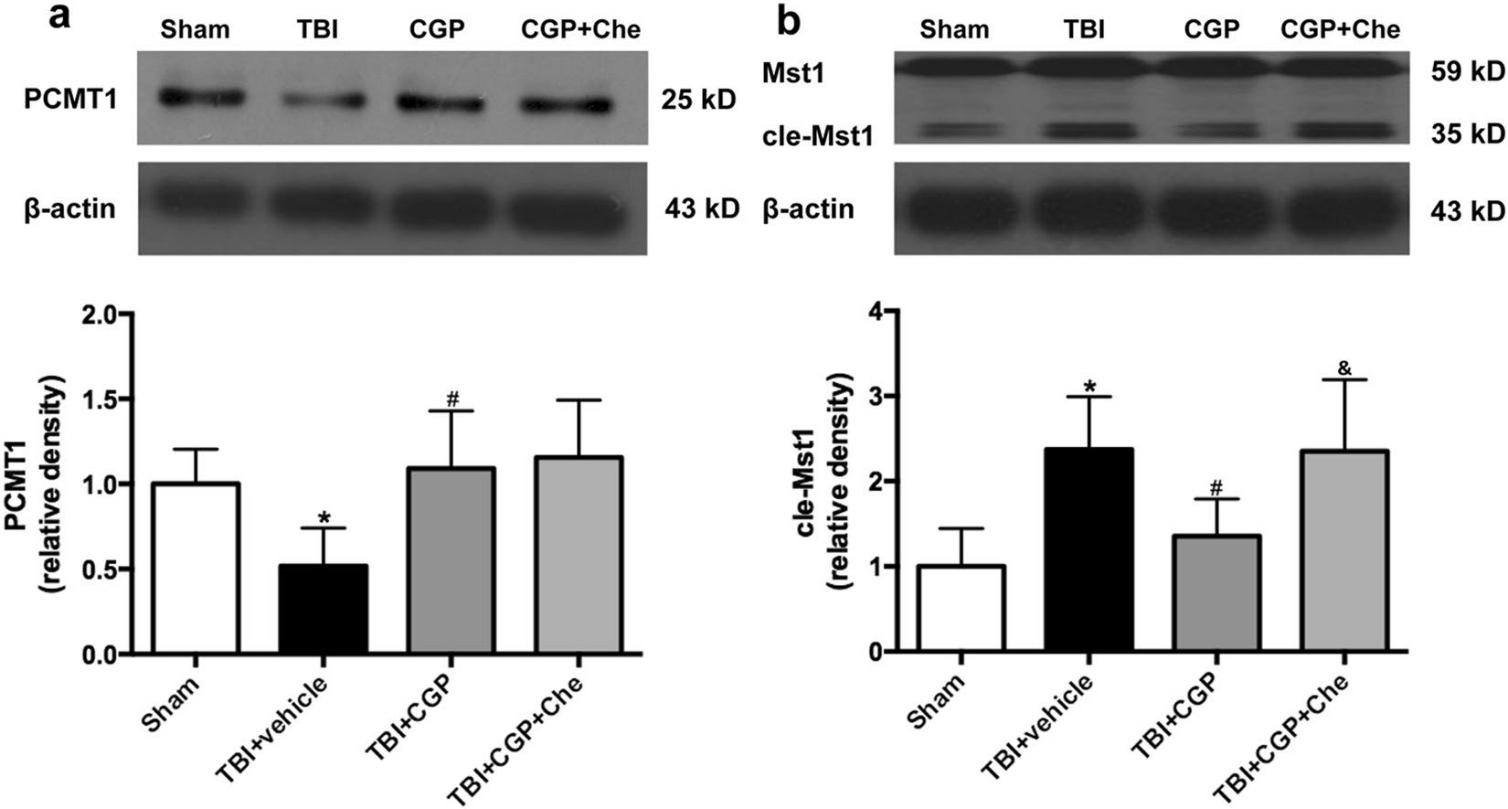
CGP3466B upregulated PCMT1 expression and inhibited cleaved-Mst1 activation at 24 h after TBI. Chelerythrine activated the Mst1 without regulating PCMT1 protein level. (**a**) Western blot assay for the expression of PCMT1. (**b**) Western blot assay for the expression of Mst1 and cleaved-Mst1. Data are represented as the mean ± SD, n = 6 rats per group. *P < 0.05 vs sham, ^#^ *P* < 0.05 vs TBI+ vehicle, ^&^ *P* < 0.05 vs TBI+ CGP. Full-length gels were displayed in supplemental materials.

Down regulation of PCMT1 was observed 24 h after TBI when compared to the sham group (*P* < 0.05, Fig. 3a). Accordingly, the protein expression level of cleeaved-Mst1 was upregulated 24 h after TBI relative to the sham group (*P* < 0.05, Fig. 3b). Treatment with CGP3466B upregulated the PCMT1 level as previously described^7, 11^ (*P* < 0.05, Fig. 3a). Simultaneously, the cleaved-Mst1 level was downregulated when compared with the TBI+ vehicle group (*P* < 0.05, Fig. 3b). The protein level of Full-length Mst1 was not changed significantly (statistical data not shown). These results suggested that Mst1 activation might be controlled by PCMT1 expression level.

### CGP3466B Regulated the Expression of Apoptosis-related Proteins After TBI

**Figure 4:**
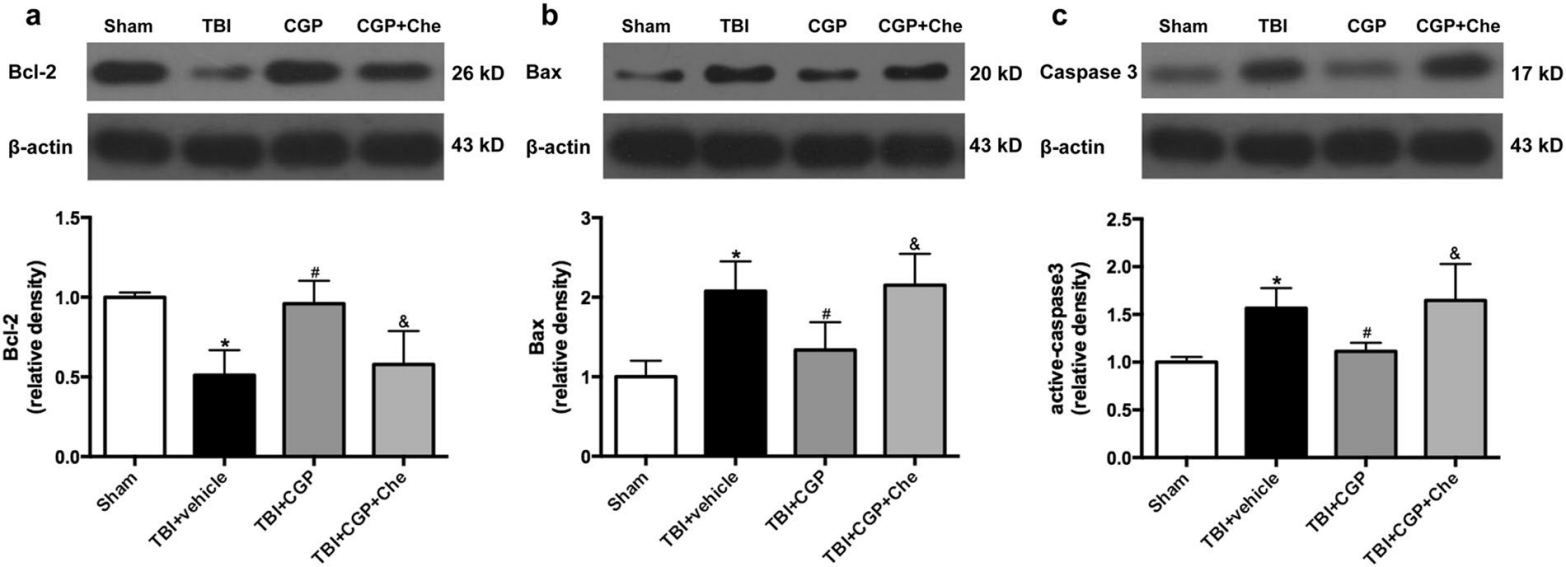
The effects of induced overexpression PCMT1 on the pro-apoptotic proteins expression at 24 h after TBI. Chelerythrine was able to reverse these effects of PCMT1 by activating the cleaved-Mst1 without changing the PCMT1 levels. (**a**) Western blot assay for the expression of Bcl-2. (**b**) Western blot assay for the expression of Bax. (**c**) Western blot assay for the expression of active-caspase3. Data are represented as the mean ± SD, n = 6 rats per group. *P < 0.05 vs sham, ^#^ *P* < 0.05 vs TBI+ vehicle, ^&^ *P* < 0.05 vs TBI+ CGP. Full-length gels were displayed in supplemental materials.

The Bcl-2 level was decreased but Bax and active-caspase3 levels were both increased 24 h after TBI (P < 0.05, Fig. 4). Treatment with CGP3466B significantly increased the protein level of Bcl-2 as well as decreased the protein levels of Bax and active-caspase3 (P < 0.05, Fig. 4). These western blot results demonstrated the anti-apoptotic effects of CGP3466B.

### CGP3466B Reduced TUNEL-positive Cells in Cortex around Injury

**Figure 5:**
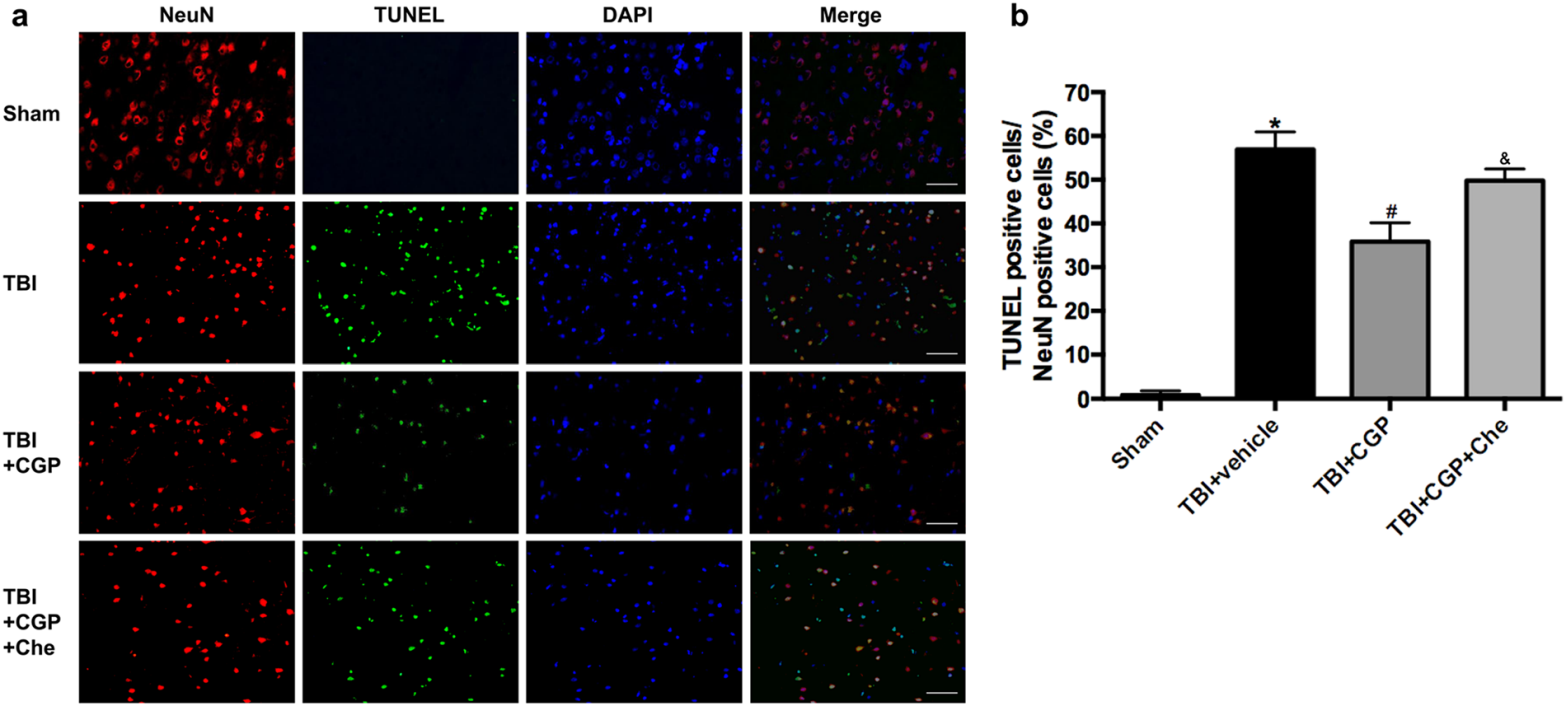
Effects of CGP3466B on cell apoptosis in the peri-injured cortex at 24 h after TBI. (**a**) Representative TUNEL/NeuN/DAPI photomicrographs of the peri-injured cortex in different groups. Fluorescence colors: TUNEL-green, NeuN-red and DAPI-blue. Scale bar = 50 μm. (**b**) Quantification of TUNEL-positive neurons in different groups, expressed as percentage of total neurons (NeuN+). Data are represented as the mean ± SD, n = 6 rats per group. *P < 0.05 vs sham, ^#^ *P* < 0.05 vs TBI+ vehicle, ^&^ *P* < 0.05 vs TBI+ CGP.

In the TBI+ vehicle group, numerous TUNEL and NeuN double-staining cells were observed when compared with the sham group (*P* < 0.05, Fig. 5). However, CGP3466B treatment was able to reduce the number of TUNEL-positive neurons significantly (*P* < 0.05, Fig. 5). These TUNEL staining results also support the anti-apoptotic effects of CGP3466B.

### Chelerythrine Abolished the Anti-apoptosis effects of CGP3466B without Regulating PCMT1 Level

Chelerythrine, as a cleaved-Mst1 activator^19^, was administered to further explore the potential mechanisms of CGP3466B neuroprotective effects. Western blot results revealed that administration of Chelerythrine was able to activate the cleaved-Mst1 (*P* < 0.05, Fig. 3b). Subsequently, the protein level of Bcl-2 was downregulated. The Bax and active-caspase3 were both upregulated significantly when compared with the TBI+ CGP group (*P* < 0.05, Fig. 4). Besides, the TUNEL staining results also showed that chelerythrine increased the number of TUNEL-positive neurons when compared with the TBI+ CGP group (*P* < 0.05, Fig. 5). However, the protein level of PCMT1 was not changed with the activation of cleaved-Mst1 in TBI+CGP+ Che group (*P* < 0.05, Fig. 3a). Besides, we also found chelerythrine can abolish the protective effect of CGP3466B on behavioral outcome. The mNSS of TBI+CGP+ Che group was significantly higher than the CGP treatment group (*P* < 0.05, data not shown).

## Discussion

TBI results in primary and secondary injury mechanisms that lead to neuronal dysfunction^3^. Secondary brain injury influences the overall outcome of patients and the neuronal apoptosis is an important contributor to secondary brain injury post-TBI^20–23^. Therefore, inhibition of neuronal apoptosis is obviously an important potential therapeutic target for the recovery following TBI.

PCMT1 is an enzyme that recognizes and repairs abnormal L-isoaspartyl residues in proteins^5^. PCMT1 overexpression was reported to ameliorate apoptosis and oxidative stress in various species and cell types^7–9, 11, 24^. Mst1 is a core member of the Hippo signaling pathway involved in a variety of regulatory mechanisms^13^. Mst1 is cleaved by caspase during apoptosis and results in increased Mst1 kinas activity, leading to apoptosis^16^. In particular, Mst1 has been suggested to mediate cell death initiated by oxidative stress in several cell types and species^11, 14, 15, 25, 26^. CGP3466B, as an anti-Parkinsonism drug, was able to upregulate PCMT1 expression in neurons and cardiomyocytes^7, 11^. In this study, we focus on the neuroprotective effects of CGP3466B and explore a possible underlying PCMT1/Mst1 neuronal anti-apoptotic pathway in a TBI rat model.

Briefly, we made the following observations in the present study: administration of CGP3466B improved neurological function, downregulated the ROS level, alleviated brain edema and inhibited neuronal apoptosis by upregulating PCMT1 expression at 24 h after TBI.

ROS are byproducts of the normal metabolism of oxygen including free radicals such as superoxide, hydroxyl radical and singlet oxygen. After TBI, endogenous antioxidants such as superoxide dismutase, glutathione peroxidase and catalase are disrupted, resulting in oxidative cell damage^27^. Excessive ROS oxidize membrane phospholipids and membrane proteins altering the structural integrity of the inner mitochondrial membrane^28^. Previous studies have showed that ROS generated by DA were able to inhibit the transcriptional activity of the human PCMT1 gene^9^. Accordingly, PCMT1 overexpression conferred antioxidant properties protecting cell components against the ROS generated by cytosolic DA^8^. The antioxidant effects of CGP3466B observed in the present study probably result from PCMT1 overexpression.

The expression level of cleaved-Mst1 was downregulated following the PCMT1 overexpression in CGP3466B treatment group. There are many downstream substrates of Mst1, which had been identified in previous studies, such as FOXO^14^, LATS1/2^29^ and JNK^30^. Considering PCMT1 overexpression and inhibited Mst1 activation following CGP3466B, we explored the downstream apoptotic proteins of Mst1 including Bcl-2 and Bax to further investigate the underlying mechanism. Bax is a known pro-apoptotic protein^31, 32^ and its overexpression has been shown to induce apoptosis in almost all cell types. The Bax expression increased and the Bcl-2 expression decreased significantly 24 h after TBI. We also examined the protein expression of active-caspase3, which triggers the cleavage of a number of proteins that ultimately lead to DNA fragmentation and apoptosis^33^. Thus, cleaved-Mst1, as the activated form of Mst1, may induce Bax expression and inhibit the Bcl-2 expression leading to caspase3 activation and cell apoptosis. TUNEL staining results also support that CGP3466B suppresses apoptosis reducing the number of TUNEL-positive cells in early brain injury after TBI. In conclusion, CGP3466B alleviates neuronal apoptosis by increasing PCMT1 expression and subsequently inhibiting MST1 activation, resulting in changing the expression levels of Bax, Bcl-2 and active-caspase3.

Results of the present study suggest that PCMT1 and Mst1 were both involved in the CGP3466B neuroprotective effects. Protein-protein interaction between PCMT1 and Mst1 was identified in HEK293 cell recently^11^. Our western blot results suggested Mst1 activation might be controlled by PCMT1 expression level. To further explore the possible relationship between PCMT1 and Mst1, we administered chelerythrine in our study, which is a known cleaved-Mst1 activator in myocytes^19^. The TUNEL staining results showed that administration of chelerythrine increased the number of TUNEL-positive cells when compared with the TBI+ CGP group at 24 h after TBI. Besides, western-blot results also revealed administration of chelerythrine upregulated cleaved-Mst1, Bax and active-caspase3 expression levels leading to neuronal apoptosis. However, the protein level of PCMT1 was not changed with the activation of cleaved-Mst1. These observations also supported that PCMT1 might be an upstream protein of Mst1.

The protein-protein interaction between PCMT1 and Mst1 requires the kinase domain of Mst1^11^. The kinase domain of Mst1 is necessary in Mst1 activation forming cleaved-Mst1. The present study suggested that Mst1 activation was controlled by PCMT1 expression level. We hypothesized that overexpressed PCMT1 proteins bind with more Mst1 than normal condition in the CGP3466B treatment group. This binding between PCMT1 and Mst1 makes only a little portion of unbound Mst1 can be activated after TBI injury, leading to less apoptosis when compared with the TBI+ vehicle group. However, in the TBI+CGP+ CHE group, chelerythrine is able to activate higher proportion of unbound Mst1 protein without changing the PCMT1 level. Therefore, the protein level of cleaved-Mst1 will be upregulated significantly by chelerythrine, even though the already bounded PCMT1-Mst1 proteins are not influenced. Then the cleaved-Mst1 activated by chelerythrine leads to more apoptosis when compared with TBI+CGP group. Additional experiments are still needed to further confirm this hypothesis and explore more specific relationship between PCMT1 and Mst1 in the future.

There are several weaknesses in our study. First, the therapeutic time window and the optimal dosage of CGP3466B treatment in TBI need to be addressed. The scecond limitation was the lack of direct evidence of an interaction between PCMT1 and MST1 in neuron. Previous coimmunoprecipitation tests have proved PCMT1 binding with MST1 in cardiomyocytes. In the present study, we only proved the co-localization of PCMT1 and Mst1 in Neuron but not gave the direct evidence of interaction between them. Third, the Bcl-2 family is one of the multiple downstream proteins related with apoptosis. Hence, it is necessary to analyze the expression of other member of Bcl-2 family in the further studies when focusing on the specific apoptosis mechanism of Mst1.

So far, the regulation of neuronal apoptosis by PCMT1/Mst1 has not been well studied, especially *in vitro*, and nothing had been investigated about PCMT1/Mst1 function in brain injury. This study is the first time to focus on a potential PCMT1/Mst1 neuronal apoptosis pathway after TBI in rats. Post-injury treatment with CGP3466B confers various beneficial effects including anti-oxidant and anti-apoptosis, via elevating PCMT1 expression and subsequent inhibition of Mst1 activation in TBI. Chelerythrine, as an Mst1 agonist, is able to reverse the anti-apoptotic effects of PCMT1. So we believed that the anti-apoptotic mechanism of PCMT1 might involve, at least in part, Mst1 pathway. PCMT1 activation and Mst1 inhibition might be a therapeutic target for inhibiting cell apoptosis following TBI. As such, CGP3466B may have a promising therapeutic potential after TBI injury.

## Material and Methods

### Animals

Adult male Sprague Dawley rats weighing 280–300 g obtained from Experimental Animal Center of Zhejiang Academy of Medicine Sciences (Hangzhou, China) were used in the present study. The rats were housed in air-filtered and temperature-controlled units with 12 h light/dark cycle. Food and water were available ad libitum. The Ethics Committee of Zhejiang University approved the study. All procedures in the present study were approved by the Institutional Animal Care and Use Committee of Zhejiang University and conformed to the guidelines of the “Principles of Laboratory Animal Care” (NIH publication No. 80-23, revised 1996).

### Study Design

Eighty-four rats were randomly assigned to four groups as follows: Sham group (n = 24), TBI+vehicle group (n = 24), TBI+CGP3466B (TBI+CGP) group (n = 24) and TBI+CGP3466B+Chelerythrine (TBI+CGP+Che) group (n = 12). Western blot, modified neurological severity score (mNss), brain water content, ROS level, terminal deoxynucleotidyl transferase-mediated deoxyuridine triphosphate nick-end labeling (TUNEL) staining and immunofluorescence were assessed 24 hours after TBI injury. Other twenty-four rats were assigned to test the side effects of drugs in sham rats.

### Drug Administration

CGP3466B (MedChem Express) was dissolved in phosphate-buffered saline (PBS, 0.1 mol/L, pH 7.4). The treatment group was injected with CGP3466B intraperitoneally at a dose of 0.14 mg/kg at 2 h after the TBI injury^34^. The TBI+ CGP+ Che group also received 10 µl of Chelerythrine (1 mmol/L, MedChem Express) intracerebroventricularly at a rate of 1 μL/min 30 min before TBI injury. The sham group and vehicle group received the same volume of PBS intraperitoneally at the same point as treatment group. All drugs have no side effects on phenotypes in sham rats (Supplemental materials).

### TBI Rat Model

The controlled cortical impact (CCI) injury model was employed as described previously (Fig. 1a)^35^. Briefly, rats were anesthetized using pentobarbital sodium (50 mg/kg intraperitoneally) and the head was mounted on a stereotaxic frame. A 5 mm diameter craniotomy between bregma and lambda over the left cortex was located following a midline incision and retraction of the skin. The skull disk was removed without damaging the dura. The CCI was performed perpendicular to the brain surface using a Benchmark CCI Stereotaxic Impactor (Benchmark Deluxe™; St. Louis, MO, USA) with a 4.0 mm diameter tip (3.0 m/s; 120 ms dwell time; 2 mm depth). The core body temperature of the rat was maintained at 36.5–37 °C during surgery using a rectal thermometer coupled to a heating pad. The skull disk was replaced and sealed immediately, and the scalp was sutured. The rats in the sham group received identical surgical procedures without CCI. All rats were put in a heated chamber to recover from anesthesia before being returned to their home cages.

### Evaluation of Neurological Deficits

Modified neurological severity score (mNSS) was used to examine the effects of overexpression PCMT1 on the neurological deficits of the animals 24 hours after TBI. Each group was blindly evaluated. The total score was ranged on a scale of 0–18, as previously described^35, 36^.

### Brain Water Content

At 24 h after TBI, the rats were sacrificed. Afterwards, the left hemispheres of brains were removed and weighed immediately to obtain the wet weight. Then the samples were baked at 105 °C for 24 hours to obtain the dry weight. The brain water content was calculated as follows: [(wet weight − dry weight)/water weight] × 100%.

### ROS Assay

The injured cortical samples were collected at 24 h after TBI. The total ROS levels were measured according to the manufacturer’s protocol with ROS assay kit (Beyotime Biotechnology). The fresh cortexes were dissected and homogenized in PBS. After centrifugation at 1000 × g for 10 min at 4 °C, the protein content of each sample’s supernatant was measured with DC protein assay kit (Bio-Rad). Afterwards, the ROS level of supernatant was measured with dichlorodihydrofluorescein diacetate (DCFH-DA) assay. The supernatant (190 μL) and 1 mmol/L DCFH-DA (10 μL) were mixed and added into 96-well plates, then incubated at 37 °C for 30 min. The mixture fluorescence intensity was measured by spectrofluorophotomery at an excitation wavelength of 480 nm and an emission wavelength of 520 nm. The ROS levels of injured cortical samples were calculated as fluorescence intensity/gram protein.

### Western Blot

The rats were sacrificed after anesthesia and the brains were quickly removed. The peri-injured cortex of TBI rats and the equivalent area in the brain of sham rats were dissected and frozen in liquid nitrogen. The cortical samples were homogenized and centrifuged at 1000 × g for 15 min at 4 °C. The resulting supernants were further centrifuged for 15 min and the protein content was measured with DC protein Assay (Bio-Rad, Hercules, CA, USA). Equal amount of protein (60 μg) from each sample was re-suspended in loading buffer and denatured at 100 °C for 5 min. Proteins were separated by 10% sodium dodecyl sulfate-polyacrylamide gel electrophoresis (SDS-PAGE), and then transferred onto polyvinylidene fluoride membranes. The membranes were blocked with nonfat dry milk for 2 h and were subsequently incubated overnight at 4 °C with polyclonal primary antibodies against PCMT1 (25kd, 1:200, #hpa003239, Atlas Antibodies, Sigma), Mst1 (59kd, 1:1000, #cst3682s, Cell Signaling Technology), cle-Mst1 (35kd, 1:1000, #cst3682s, Cell Signaling Technology), Bax (20kd, 1:1000, #cst14796, Cell Signaling Technology), Bcl-2 (26kd, 1:1000, #ab194583, Abcam), active-caspase3 (17kd, 1:500, #ab49822, Abcam) and β-actin (43kd, 1:2000, #sc47778, Santa Cruz). Then the membranes were incubated with HRP-linked secondary antibodies for 1 h at room temperature. Then membranes were washed and visualized on by enhanced chemiluminescence and X-ray film. Quantitative analysis was conducted with ImageJ software (National Institutes of Health, Bethesda, MA, USA).

### Immunohistochemistry and Quantification of Neuronal Cell Death

To evaluate co-localization of neurons and Mst1/PCMT1, double staining for neuron-specific nuclear protein (NeuN) and Mst1/PCMT1 were performed. The rats were sacrificed and perfused intracardially with PBS and 4% paraformaldehyde. Brains were collected and immersed in 4% paraformaldehyde at 4 °C for 12 h, followed by immersion in 30% sucrose solution until the tissue sank. Coronal frozen sections (10 um) were created for fluorescence staining. The brain sections were washed with 0.1 mol/L PBS and incubated with blocking solution (10% normal donkey serum) at room temperature for 1 hour. Then the sections were rinsed with 0.1 mol/L PBS and incubated at 4 °C overnight with primary antibodies including mouse anti-Mst1 (1:250, #611052, BD Bioscience), rabbit anti-NeuN (1:250, #ab177487, Abcam), rabbit anti-PCMT1 (1:250, #hpa003239, Atlas Antibodies, Sigma) and mouse anti-NeuN (1:250, #ab104224, Abcam). Second day, the sections were rinsed again and incubated with secondary antibodies at 37 °C for 2 hours. Finally, the sections were mounted with the Fluoroshield with DAPI (Sigma) and observed under a fluorescence microscope (Leica).

Double label staining of TUNEL with NeuN was performed to co-localization of apoptotic neurons. The section was stained with the antibody against NeuN (1:250, Abcam), then the TUNEL staining was performed according to the manufacturer’s protocol (Roche Inc, Basel, Switzerland). Finally, the sections were covered with DAPI and observed under a fluorescence microscope (Leica).

### Statistical Analysis

All data were expressed as the mean ± standard deviation (SD) and analyzed with SPSS 19.0 statistical software. One-way analysis of variance (ANOVA) followed by Turkey’s multiple comparison tests was performed for multiple comparisons. Statistical significance was defined as *P* < 0.05.

### Compliance with ethical standards

All experimental protocols involving animals (including all surgical procedure) were approved by the Institutional Animal Care and Use Committee (IACUC) of the Zhejiang University. All rat experimental procedures were performed in accordance with the Regulations for the Administration of Affairs Concerning Experimental Animals approved by the State Council of People’s Republic of China.

## Electronic supplementary material

Supplementary Materials
