# Supplementary material for: Neuroprotective Effects of CGP3466B on Apoptosis Are Modulated by Protein-L-isoaspartate (D-aspartate) O-methyltransferase/Mst1 Pathways after Traumatic Brain Injury in Rats
Source: Sci Rep. 2017 Aug 23;7:9201. doi: 10.1038/s41598-017-08196-3 (PMC5569064; doi:10.1038/s41598-017-08196-3)

Neuroprotective Effects of CGP3466B on Apoptosis Are Modulated by Protein-L-isoaspartate (D-aspartate) O-methyltransferase/Mst1 Pathways after Traumatic Brain Injury in Rats

Feng Liang[1*], Ligen Shi[1*], Jingwei Zheng[1], Sheng Chen[1], Yangxin Wang[2], Jianmin Zhang[1]

1Department of Neurosurgery, Second Affiliated Hospital, School of Medicine, Zhejiang University, Hangzhou 310009, China

2 Department of Orthopaedics, Second Affiliated Hospital, School of Medicine, Zhejiang University, Hangzhou 310009, China

*The first two authors contributed equally to this work

Correspondence author: Jianmin Zhang, Email: zjm135@vip.sina.com, Tel: +860571-86095135

Effects of CGP3466B and chelerythrine on neurological function and brain edema in sham rats. (a) Quantification of the mNSS. (b) Quantification of the brain water content. n=6 rats per group. ****P*< 0.05 vs sham.**

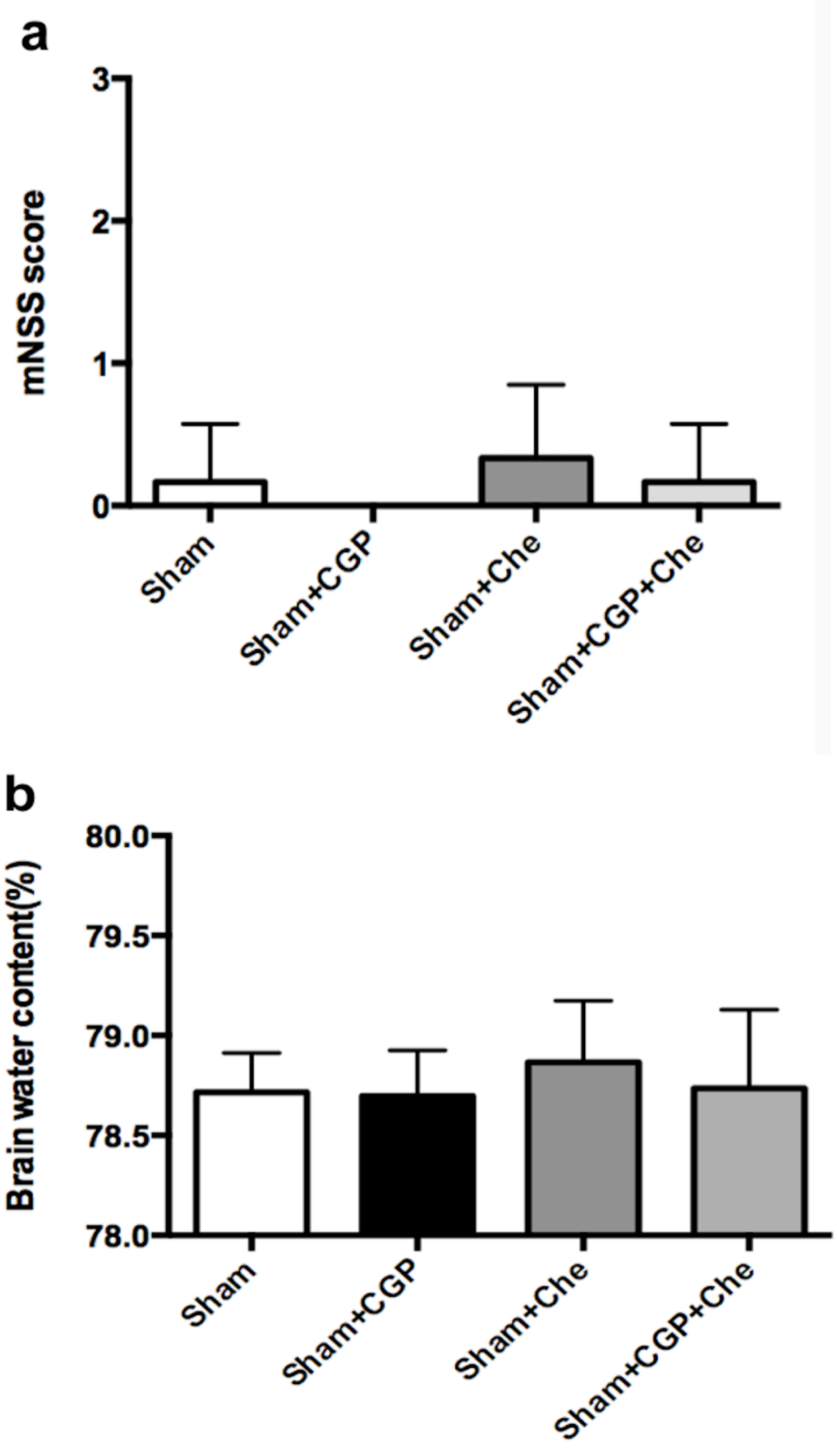

Full-length gels for Figure 3

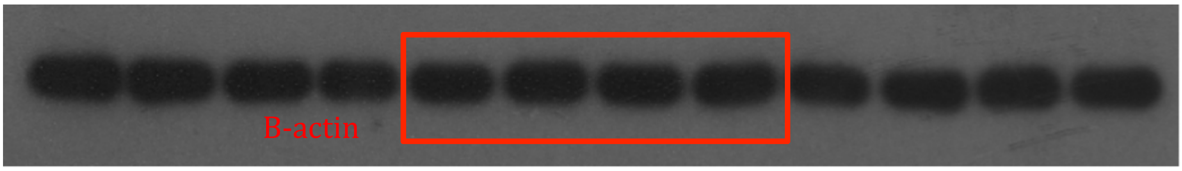

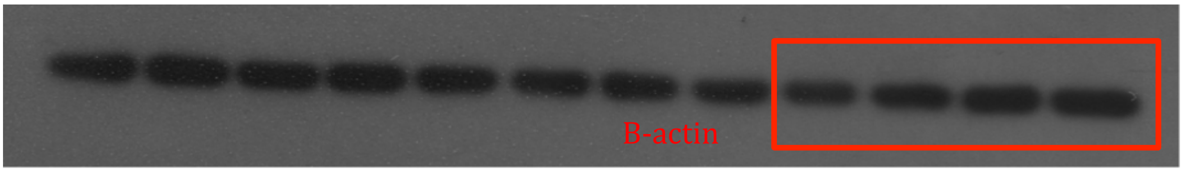

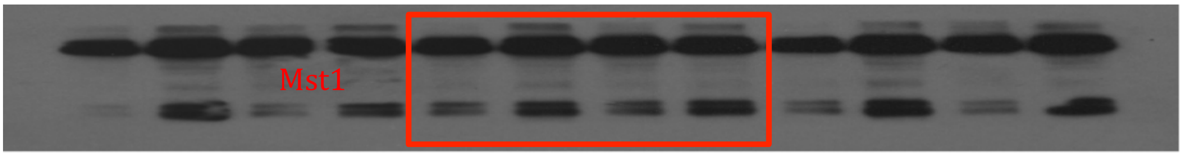

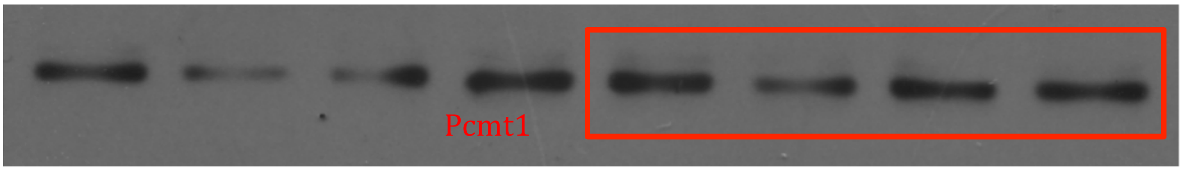

Full-length gels for Figure 4

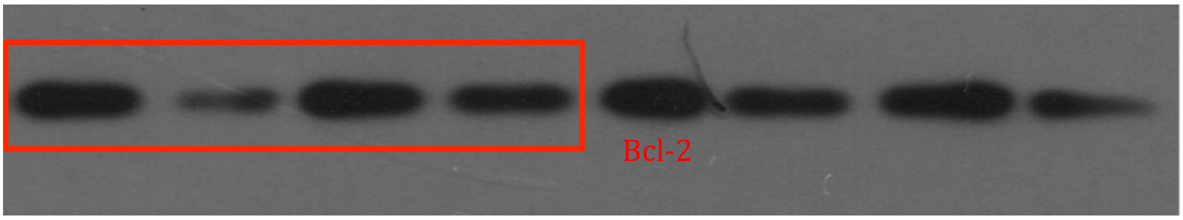

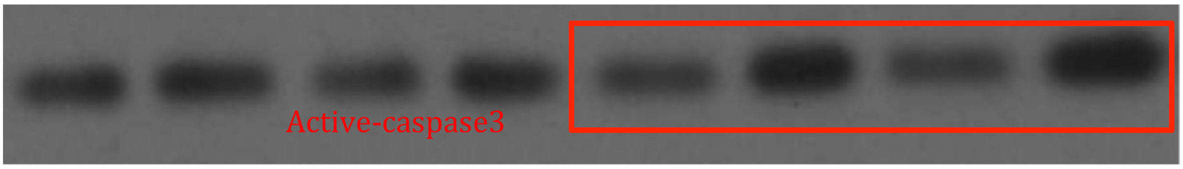

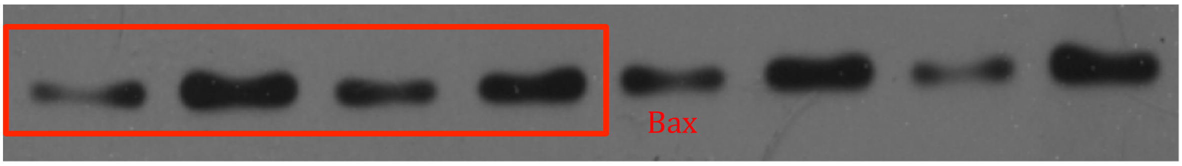

Supplement: Supplementary file 1 — Supplementary Materials [file 41598_2017_8196_MOESM1_ESM.pdf]
